# The Importance of Timing in Performing a Holter ECG in Patients Diagnosed with an Embolic Stroke of Undetermined Source

**DOI:** 10.3390/biomedicines13040771

**Published:** 2025-03-21

**Authors:** Dia Alwilly, Saher Srour, Irina Nordkin, Asaf Honig, Karine Beiruti Wiegler, Ronen R. Leker, Naaem Simaan

**Affiliations:** 1 Department of Neurology, Ziv Medical Center, Safed 1311001, Israel; dia.alwilly@gmail.com; 2 Department of Radiology, Ziv Medical Center, Safed 1311001, Israel; sahers@ziv.gov.il; 3 Department of Cardiology, Ziv Medical Center, Safed 1311001, Israel; irinan@ziv.gov.il; 4 Department of Neurology, Soroka Medical Center, Beer-Sheva 8410100, Israel; asaf.honig2@gmail.com; 5 Faculty of Health Science, Ben Gurion University, Beer-Sheva 8410501, Israel; 6 Research Wing, Ziv Medical Center, Safed 1311001, Israel; karinebeiruti@ziv.gov.il; 7 Department of Neurology, Hadassah-Hebrew University Medical Center, Jerusalem 9112000, Israel; leker@hadassah.org.il; 8 Azrieli Faculty of Medicine, Bar Ilan University, Safed 1311502, Israel

**Keywords:** previously undiagnosed atrial fibrillation, embolic stroke of undetermined source, Holter ECG

## Abstract

**Background/Objectives**: Previously undiagnosed atrial fibrillation (PUAF) is a significant cause of embolic stroke of undetermined source (ESUS). This study aimed to determine whether early heart rhythm monitoring with a Holter ECG after acute stroke enhances the detection of PUAF compared to standard ambulatory monitoring in ESUS patients, assuming that early cardiac monitoring would lead to a higher detection rate of PUAF. **Methods**: This cohort study included 100 patients aged 50 and older diagnosed with ESUS and exhibiting sinus rhythm for at least 24 h. All participants were hospitalized in a stroke unit and underwent 48 h of Holter ECG monitoring. A group of 100 ESUS patients who underwent outpatient delayed Holter ECG monitoring served as controls. **Results**: This study revealed a significantly higher detection rate of AF in the hospitalized group compared to the outpatient group (20% vs. 5%; *p* = 0.001). The mean age and distribution of risk factors, including hypertension, diabetes, hyperlipidemia, ischemic heart disease, heart failure, chronic kidney disease, smoking, previous stroke, and malignancy, did not differ between the groups. There were no significant differences in initial stroke severity or in outcomes between the groups. **Conclusions**: Early Holter ECG monitoring in the hospitalized ESUS patients significantly increased the detection rate of PUAF compared to ambulatory monitoring, highlighting the importance of timely cardiac assessment in stroke management.

## 1. Introduction

Ischemic stroke remains a leading cause of morbidity and mortality worldwide, affecting millions of individuals annually. Approximately 25% of ischemic stroke cases are classified as cryptogenic, or of undetermined origin, despite recommended extensive testing [1,2]. Among these cases, stroke imaging suggests an embolic cause, yet no identifiable source can be found, leading to the classification of embolic stroke of undetermined source (ESUS) [3,4]. This subgroup of ischemic stroke presents a significant diagnostic challenge, as the inability to pinpoint the underlying etiology complicates treatment decisions and makes effective risk stratification difficult. Consequently, patients with ESUS are often left without a targeted therapeutic approach, which can lead to suboptimal outcomes [5,6].

ESUS is thought to result from various potential etiologies, some of which may not be immediately apparent or detectable through standard diagnostic techniques. Frequent underlying causes include previously undiagnosed atrial fibrillation (PUAF), non-stenotic vulnerable atherosclerotic plaques, patent foramen ovale (PFO), occult malignancies, and hypercoagulable disorders [6,7,8]. Among these, due to its intermittent nature, PUAF remains particularly elusive to detect, despite being a well-established risk factor for embolic stroke. Given the high prevalence of cryptogenic stroke, the ability to identify PUAF early in the diagnostic process is critical, as it could significantly influence both the immediate management and the long-term outcomes for patients [9].

Current diagnostic techniques for detecting atrial fibrillation (AF), such as standard 12-lead electrocardiogram (ECG) monitoring, have inherent limitations in detecting short episodes of AF, particularly when the arrhythmia is paroxysmal or intermittent. To address this limitation, several advanced diagnostic tools and wearable devices have been developed, including serial ECG monitoring, 24–48 h Holter monitoring, external loop recorders, implantable cardiac devices (CIEDs), and implantable cardiac monitors (ICMs) [10,11,12]. While these devices have proven effective in detecting intermittent AF, the optimal timing and method for their application in ESUS patients remain unclear. Specifically, it remains unclear whether initiating detection early, during the acute phase of a stroke, via tools such as Holter monitoring would provide more conclusive findings compared to the traditional practice of outpatient monitoring, which is generally performed after the acute phase [13].

It has been hypothesized that the early detection of PUAF during the acute phase of a stroke through techniques like Holter monitoring may allow for the prompt initiation of preventive therapies, such as direct oral anticoagulants (DOACs). The early initiation of such therapies could potentially reduce the risk of recurrent ischemic strokes and improve the long-term functional outcomes for ESUS patients. However, despite its potential benefits, the current evidence regarding the most effective timing and modality of PUAF monitoring in ESUS patients remains inconclusive. To address this gap, the aim of this study is to examine and compare the diagnostic yield of early Holter monitoring during the acute phase of stroke with traditional outpatient monitoring. By evaluating whether the early identification of PUAF could lead to more accurate stroke management and better patient outcomes, this study aims to optimize the diagnostic process for this challenging, high-risk patient population.

## 2. Materials and Methods

This study involved 100 patients aged 50 and older, all of whom were admitted to the stroke unit of Ziv Medical Center (ZMC) between January 2021 and January 2023. The study focused on patients diagnosed with embolic stroke of undetermined source (ESUS), which was defined according to the criteria established by Hart et al. [4]. According to these criteria, ESUS is characterized by a non-lacunar ischemic stroke, as identified by either CT or MRI imaging, where there is no evidence of extra- or intracranial atherosclerosis causing ≥50% luminal stenosis in the relevant arteries. Additionally, a cardioembolic source or any specific stroke causes, such as arterial dissection, migraine, vasospasm, or drug abuse, must be absent.

All ESUS diagnoses underwent validation by a vascular neurologist to maintain the diagnostic accuracy, following a comprehensive and structured etiological evaluation. This evaluation process included a thorough assessment consisting of a 12-lead electrocardiogram (ECG) and transthoracic echocardiography; furthermore, all patients underwent 24 h cardiac monitoring with automated rhythm detection during the first day of admission to the stroke unit, and imaging of the extracranial and intracranial arteries supplying the ischemic region through MR or CT angiography techniques. These diagnostic procedures were critical for ruling out the common stroke etiologies and confirming the diagnosis of ESUS in each participant.

All participants in the study presented with sinus rhythm and had no prior history of atrial fibrillation (AF). Furthermore, all patients underwent 24 h cardiac monitoring with automated rhythm detection on the first day of admission to the stroke unit, with no evidence of atrial fibrillation observed during the 24 h monitoring period. Furthermore, the inclusion criteria required that the participants had ischemic lesions larger than 3 cm, as confirmed by MRI, and that they were admitted to the emergency department within 24 h of stroke onset. These criteria helped in ensuring that the study population consisted of patients experiencing acute ischemic strokes, thus providing valuable insight into the early-phase management and diagnostic evaluation of ESUS.

After 24 h of continuous cardiac monitoring without any evidence of atrial fibrillation (AF) detection, all patients underwent Holter ECG monitoring for a minimum of 48 h during hospitalization. This extended monitoring was implemented to assess the presence of paroxysmal or intermittent AF that might not have been captured during the initial 24 h monitoring period. An independent cardiologist, blinded to the clinical details, assessed all ECG recordings for rhythm variations. Each patient underwent a 48 h, 3-channel ECG evaluation. Paroxysmal AF (PAF) was defined as chaotic atrial activity without distinct P waves and irregular RR intervals lasting at least 30 s, following a manual review to eliminate artifacts and classification errors.

The diagnoses and evaluations were conducted by senior neurologists, ensuring expert interpretation of the clinical findings and diagnostic tests.

The results of this cohort were then compared to a control group of 100 ESUS patients who underwent ambulatory Holter monitoring. This comparison allowed for an assessment of the diagnostic yield of the early Holter monitoring during hospitalization versus the traditional outpatient monitoring method, providing further insight into the potential advantages and limitations of each approach. By examining the diagnostic performance of these two monitoring strategies, this study aimed to determine the value of the early detection of atrial fibrillation in patients with ESUS and its implications for stroke management and prevention.

The exclusion criteria included an age of less than 50 years, lacunar infarcts, rheumatic valvular disease, dilated cardiomyopathy, prior AF or rhythm abnormalities at admission, prosthetic heart valves, and significant hemodynamic narrowing of the ipsilateral internal carotid arteries as determined by CTA.

The data were collected in the anonymized registry of the stroke unit, maintained by trained vascular neurologists. For each patient, the demographic information, medical history, treatment regimens, and initial clinico-radiologic characteristics were documented. The clinical stroke severity was assessed using the National Institutes of Health Stroke Scale (NIHSS). Instances of symptomatic arterial occlusion were identified by CT or Magnetic Resonance Angiography (MRA), and the use of revascularization treatments (IV thrombolysis or mechanical thrombectomy) was recorded. The cardiac evaluations included morphological, electrophysiological, and biochemical assessments. A trained cardiologist reviewed the 48 h Holter ECG recordings.

The collected data were anonymized and securely stored in an Excel file on the department’s drive, labeled with the study title and Helsinki approval number.

Statistical analysis: Data analysis was performed with SPSS version 25 (SPSS Inc., Chicago, IL, USA), and descriptive statistics were used to summarize the data. Normally distributed continuous variables were reported as means with standard deviations, while non-normally distributed variables were presented as medians. Qualitative variables were analyzed using frequency counts and means. *t*-tests were used for the normally distributed continuous variables, while non-parametric tests were employed for the discrete variables. Chi-square tests assessed differences in the clinical and demographic variables between the groups. The results were summarized in frequency tables, and a *p*-value of 5% or less was considered statistically significant.

The study was conducted in accordance with the Declaration of Helsinki and approved on 4 April 2023 by Ethics Committee ZIV 0129-22.

## 3. Results

One hundred patients diagnosed with ESUS (embolic stroke of undetermined source), aged 50 and older with sinus rhythm for at least 24 h of monitoring in the stroke unit and no previous history of atrial fibrillation, were included in this prospective database that was retrospectively analyzed. The results were compared with 100 patients with ESUS who underwent ambulatory Holter ECG monitoring after ESUS. Table 1 presents the demographic and clinical characteristics of the study patients according to hospitalization type—the hospitalized ESUS patients versus those who underwent ambulatory Holter ECG.

The mean age of the patients was similar across all groups; there were higher proportions of males in both groups, 58.5% among the hospitalized patients and 68.6% among the ambulatory patients, though the difference was not statistically significant. Common risk factors such as hypertension, diabetes, hyperlipidemia, ischemic heart disease, heart failure, chronic kidney disease, smoking, prior stroke, and malignancy showed no significant differences between the hospitalized and ambulatory groups. Statin use was significantly higher in the ambulatory patients (76%), with 58% of the hospitalized patients receiving statin therapy (*p* = 0.007). Other treatments, such as Coumadin, DOACs, and t-PA, showed no significant differences between the two groups.

The characteristics and outcomes of the stroke patients, comparing the early Holter monitoring at admission with the conventional outpatient Holter monitoring, are represented in Table 2.

The ambulatory patients had a longer median time from stroke onset to Holter monitoring compared to the hospitalized patients, 53 days versus 3 days (*p* < 0.001). No significant difference was observed in the NIHSS scores at admission, with a median score of five in both groups. Similarly, no significant difference was found in the NIHSS disability scores between the groups. There were no significant differences in the MRS scores before stroke and at discharge between the hospitalized and ambulatory groups. The distribution of right, left, or bilateral strokes was similar between the two groups. Overall, while there were slight variations in the vascular lesion distribution between the hospitalized and ambulatory patients, these differences were not statistically significant (*p* > 0.05). The majority of the patients in both groups were discharged directly home, with no significant difference between both groups. There was no significant difference in the mortality rates between the hospitalized and ambulatory groups. The occurrence of new atrial fibrillation was significantly higher among the hospitalized patients at 20%, compared to 5% in the ambulatory group (*p* = 0.001).

## 4. Discussion

The observation of a significantly higher rate of post-acute atrial fibrillation (PUAF) in the hospitalized group compared to the ambulatory group is a crucial finding, with important clinical implications. This observation underscores the critical importance of early cardiac monitoring after an embolic stroke of undetermined source (ESUS). ESUS represents a stroke for which no clear cause can be identified despite thorough diagnostic evaluation, and it is becoming increasingly recognized that atrial fibrillation (AF) is a potential underlying cause in many of these cases. The fact that a higher rate of PUAF was detected in the hospitalized group suggests that closer monitoring during the acute phase of stroke may be essential for identifying transient or occult AF episodes that could otherwise go undetected. However, it is important to note that the acute phase, characterized by the patient’s critical condition, might also unmask AF episodes that are not clinically significant, and could be more related to the critical state of the hospitalization rather than a true underlying arrhythmia.

Previous studies indicate that the rate of newly identified AF in post-ischemic stroke varies based on the definitions, study populations, monitoring duration, devices (e.g., Holter ECG, external loop recorders), and the timing of monitoring initiation [14]. The current guidelines recommend a minimum of 24 h of ECG monitoring after a stroke [15], underscoring a gap in the evidence regarding the use of adequate monitoring techniques. Approximately 25% of ischemic strokes are classified as cryptogenic, with increasing evidence suggesting that PUAF is an important cause in such cases [2,16].

The current diagnostic criteria for ESUS necessitate at least 24 h of Holter monitoring [4]. The early detection of AF is crucial for reducing the risk of recurrent ischemic strokes, as the timely initiation of anticoagulation can significantly mitigate this risk [9].

Previous studies demonstrate that continuous Holter ECG monitoring improves AF detection rates. For instance, using ambulatory Holter monitoring for 1 to 7 days yielded a detection rate of 10.7%, which increased to 15% with monitoring for 7 days or more [17,18]. A recent meta-analysis of randomized controlled trials and prospective cohort studies found that implantable loop recorders (ILRs) detected AF in 4.9% of patients at 1 month, with the detection rate increasing to 38.4% at 36 months [19].

Notably, our detection rate of 20% is higher than in previous reports, likely due to the 48 h Holter ECG conducted during hospitalization, which may have effectively identified patients with occult AF. In the CRYSTAL AF trial, implantable monitors identified AF in 8.9% of patients within 6 months and 30.0% by 3 years, compared to traditional monitoring methods [20], but unlike our study, they faced delays in randomization (up to 75 days after symptom onset). This may have influenced their low detection rates. A more recent randomized clinical trial involving patients with ischemic stroke found that after 12 months, 15.3% of patients monitored with an ICM were diagnosed with AF, compared to 4.7% in the group using prolonged external loop recorders [21]. Not all AF detected after a stroke is relevant. The optimal time period of AF detection after ischemic stroke and transient ischemic attack has been highlighted in a recent systemic review and meta-analysis of randomized controlled trials, revealing two critical time periods of detection: 0–14 days after an event is crucial for identifying AF that may occur immediately after a stroke, and 181–365 days post-stroke is a significant window for capturing AF that develops months after the initial event [22]. These findings suggest that both short-term and extended monitoring protocols are essential to effectively detect AF. This approach aligns with the understanding that AF can emerge at various times post-stroke, necessitating comprehensive monitoring strategies [22]. Unlike our study, which specifically focused on ESUS patients, Thakur et al. [22] include a broader population of stroke patients.

Although 48 h Holter monitoring may have lower sensitivity than implantable monitors, it offers non-invasive and cost-effective advantages. In addition, our device can detect transient AF episodes lasting seconds, while implantable monitors often require at least a 2 min episode for detection [23]. Short AF episodes are relatively common, as evidenced by systematic reviews indicating that many recently diagnosed stroke patients with AF experience episodes lasting only 30 s [24,25]. The relationship between the role of the AF pattern (paroxysmal, persistent, or permanent) and the risk of stroke is currently a controversial subject, and the analysis is complicated by several factors, such as age, comorbidities, and concomitant medications [26]. In the current study, five of our patients exhibited AF lasting less than 2 min as their only AF episode.

The practical advantages of early monitoring in patients with ischemic stroke, particularly those with embolic stroke of undetermined source (ESUS), include the ability to make immediate and informed therapeutic decisions based on the real-time data. This is particularly important given the time-sensitive nature of stroke management, where early intervention can significantly impact patient outcomes. The ability to identify atrial fibrillation (AF) shortly after a stroke allows clinicians to begin appropriate treatments, such as anticoagulation therapy, and to reduce the risk of recurrent ischemic strokes. Studies suggest that AF detection rates decline the longer the interval between the stroke onset and the initiation of cardiac monitoring [7]. Early detection is crucial, as transient or paroxysmal AF episodes may resolve or become less frequent over time, thus missing the therapeutic window for initiating anticoagulation therapy. Therefore, early monitoring not only increases the likelihood of detecting AF, but also allows for timely interventions that may substantially reduce the risk of further strokes, Furthermore, the early detection of atrial fibrillation (AF) can significantly assist both physicians and patients by providing a clear and identifiable cause for the stroke, thus eliminating the need to pursue extensive and often unnecessary investigations for other potential etiologies of embolic stroke of undetermined source (ESUS). This can lead to a more focused diagnostic approach, where the presence of AF as a contributing factor allows for targeted treatment strategies, such as anticoagulation therapy, rather than broad and invasive diagnostic procedures. As a result, the need for further, potentially costly and invasive tests—such as additional imaging, cardiac catheterization, or even more complex procedures like transesophageal echocardiograms and the use of contrast agents and radiation exposure, can be significantly reduced or avoided. Not only does this help streamline the diagnostic process, but it also minimizes patient discomfort, risk, and healthcare costs, making the management of cryptogenic stroke more efficient and less burdensome for both patients and healthcare systems.

While extended monitoring tools, such as implantable loop recorders (ILRs), offer enhanced detection capabilities for identifying atrial fibrillation (AF), early Holter monitoring for 48 h remains a viable alternative due to its non-invasive nature, ease of application, and significant diagnostic yield. Holter monitoring is a widely accessible and established method that provides continuous ECG data over a relatively short period, making it particularly valuable in the acute phase of stroke management. Its non-invasive nature and ability to capture transient or paroxysmal episodes of AF within the first few days following a stroke make it a practical and efficient choice for initial screening, especially in settings with limited access to more advanced technologies like ILRs.

In addition, practical limitations exist, and may hinder the widespread adoption of extended monitoring tools. One key limitation is the availability of resources, as extended monitoring methods, including ILRs and longer durations of Holter monitoring, may not be feasible in all stroke units, particularly those with limited staffing or financial resources. Additionally, despite the potential for improved detection, extended monitoring tools come with their own challenges, such as the risk of false positives in ECG interpretations, which can lead to unnecessary treatments or diagnostic procedures. False positives can be particularly problematic when assessing patients who may have other underlying conditions or arrhythmias that could mimic AF on an ECG.

Despite these challenges, prioritizing early and continuous monitoring for patients with ESUS, in our opinion, is essential for the effective detection of occult atrial fibrillation and the reduction of recurrent stroke risk. The timely identification of AF in ESUS patients allows for the earlier initiation of appropriate interventions, such as anticoagulation therapy, which can substantially decrease the likelihood of subsequent strokes. While the practical limitations of extended monitoring tools should not be overlooked, integrating early and consistent monitoring strategies into stroke management protocols is crucial for improving the long-term outcomes in this patient population.

Statin use differed significantly between the two patient groups, with 76% of the ambulatory patients and 58% of the hospitalized patients receiving prior statin therapy (*p* = 0.007). This difference can likely be attributed to the continuation of statin therapy, which was most likely initiated in the ambulatory patient group following discharge from the stroke unit. In contrast, the acutely hospitalized patients had not yet begun statin therapy during their hospitalization. This observation underscores a potential gap in treatment between acute care and long-term management, highlighting the critical need for the consistent continuation of medications, such as statins, after discharge. Ensuring the proper initiation and maintenance of such therapies in the post-discharge phase is vital for improving patient outcomes and reducing the risk of recurrent strokes.

Additionally, the ambulatory patients had a significantly longer median time from stroke onset to Holter monitoring compared to the hospitalized patients (53 days vs. 3 days, *p* < 0.001). This substantial difference likely reflects the nature of the care setting, where ambulatory patients are monitored in an outpatient setting, allowing for a more delayed follow-up, while hospitalized patients are generally subjected to more immediate diagnostic procedures due to the acute nature of their condition. The delay in monitoring for ambulatory patients could have implications for early detection and intervention, as prolonged gaps between stroke occurrence and diagnostic testing may lead to missed opportunities for timely therapeutic measures.

There were no significant differences in the National Institutes of Health Stroke Scale (NIHSS) or Modified Rankin Scale (MRS) scores between the two groups at either admission or discharge.

### Limitations

The main limitations of this study are its single-center design and the small sample size, which may attenuate the statistical validity and accuracy for detecting the differences between groups and may restrict the generalizability of the findings to broader patient populations or healthcare systems. Future studies with larger, more diverse sample sizes would provide more reliable and generalizable conclusions. While the single-center approach allowed for a focused examination of the specific cohort, the results may not fully represent the diversity of the treatment approaches, demographic factors, or stroke characteristics seen in larger, multi-center studies. However, this study does have several notable strengths, including the use of a large, relatively accurate dataset and the prospective collection of the data by a hospital physician who was familiar with the documentation practices. This careful data collection process helps ensure the reliability and consistency of the findings, providing a solid foundation for further research in this area.

## 5. Conclusions

Early cardiac monitoring after ESUS can increase the yield of PUAF detection fourfold compared to standard outpatient cardiac monitoring. This early detection is critical for the timely intervention and prevention of subsequent ischemic strokes. Future guidelines should emphasize the role of early, intensive monitoring in stroke management to improve patient outcomes.

## Figures and Tables

**Table 1:** Demographic and clinical characteristics of study patients according to type of hospitalization.

Characteristics	All (n = 200)	Hospital (n = 100)	Ambulatory (n = 100)	*p*-Value
Age (mean ± SD)	68.8 (±10.6)	69.6 (±11.1)	68.0 (±10)	0.291
Gender, male (%)	126 (63.0)	58 (58.0)	68 (68.0)	0.143
Risk factors				
Hypertension (%)	132 (66.0)	63 (63.0)	69 (69.0)	0.370
Diabetes mellitus (%)	94 (47.0)	44 (44.0)	50 (50.0)	0.395
Hyperlipidemia (%)	129 (64.5)	61 (61.0)	68 (68.0)	0.301
Ischemic heart disease (%)	60 (30.0)	35 (35.0)	25 (25.0)	0.123
Congestive heart failure (%)	15 (7.5)	11 (11.0)	4 (4.0)	0.060
Chronic renal failure (%)	22 (11.0)	12 (12.0)	10 (10.0)	0.651
Smoking (%)	72 (36.0)	38 (38.0)	34 (34.0)	0.556
Previous stroke (%)	44 (22.1)	20 (20.0)	24 (24.0)	0.519
Malignancy (%)	18 (9.0)	9 (9.0)	9 (9.0)	0.999
Aspirine (n, %)	1 (0.5)	0 (0)	1 (1.0)	0.316
APLA (n, %)	2 (1.0)	1 (1.0)	1 (1.0)	0.999
Medical treatment				
Statins	134 (67.0)	58 (58.0)	76 (76.0)	**0.007**
Coumadin	2 (1.0)	1 (1.0)	1 (1.0)	0.999
NOACs	5 (2.5)	4 (4.0)	1 (1.0)	0.174
tPA (%)	38 (19.0)	24 (24.0)	14 (14.0)	0.071

APLA—antiphospholipid antibody, NOACs—novel oral anticoagulants, tPA—tissue plasminogen activator. Bold indicates significant *p*-values.

**Table 2:** Characteristics and outcomes of stroke patients, comparing those treated in hospital setting versus those receiving ambulatory care.

Variables	All (n = 200)	Hospital (n = 100)	Ambulatory (n = 100)	*p*-Value
Holter connection time, days from stroke (median, IQR)	18 (3–53)	3 (2–4)	53 (44–60)	**<0.001**
NIHSS at admission (median, IQR)	4 (3–7)	5 (2.5–7)	4 (3–7)	0.312
NIHSS, DIS (median, IQR)	2 (1–5)	2 (1–6)	3 (1–4.5)	0.533
MRS, pre-admission (median, IQR)	2 (1–4)	2 (1–4)	2 (1–3)	0.424
MRS, DIS (median, IQR)	1 (1–3)	1 (1–4)	1 (1–3)	0.610
Side (%):				
Right	66 (33.0)	33 (33.0)	33 (33.0)	0.745
Left	96 (48.0)	50 (50.0)	46 (46.0)	
Bilateral	38 (19.0)	17 (17.0)	21 (21.0)	
Vessel lesion (%):				
ICA	46 (23.0)	24 (24.0)	22 (22.0)	0.288
MCA M1	27 (13.5)	12 (12.0)	15 (15.0)	
MCA M2	52 (26.0)	30 (30.0)	22 (22.0)	
Vert	24 (12.0)	10 (10.0)	14 (14.0)	
Basilar	10 (5.0)	4 (4.0)	6 (6.0)	
ACA	15 (7.5)	4 (4.0)	11 (11.0)	
PCA	26 (13.0)	16 (16.0)	10 (10.0)	
Discharge status (%):				
Home	150 (75.0)	73 (73.0)	77 (77.0)	0.514
Rehabilitation facility	50 (25.0)	27 (27.0)	33 (33.0)	
New atrial fibrillation (%)	25 (12.5)	20 (20.0)	5 (5.0)	**0.001**
Last known status, dead (%)	1 (0.5)	0 (0)	1 (1.0)	0.316

NIHSS—National Institutes of Health Stroke Scale, MRS—Modified Rankin Scale for Neurologic Disability, DIS—discharge, IQR—interquartile range, ICA—internal carotid artery, MCA—middle cerebral artery, Vert—vertebral, ACA—anterior cerebral artery, PCA—posterior cerebral artery. Bold indicates significant *p*-values.

## Data Availability

Full data are available following a formal request and in compliance with state regulations.
